# An Assessment of the Potential Benefits of Video Consultation in the Emergency Department: Mixed Methods Study

**DOI:** 10.2196/36081

**Published:** 2022-09-15

**Authors:** Jane Turner, Malcolm Clarke, Grizelda George, Russell Wynn Jones, Rick Pullinger, Rajesh Kharbanda, James Kennedy, Linda Hands

**Affiliations:** 1 Nuffield Department of Surgical Science University of Oxford Oxford United Kingdom; 2 Department of Electronic Engineering Maynooth International Engineering College Fuzhou China; 3 Oxford University Hospitals National Health Service Foundation Trust Oxford United Kingdom; 4 Chorleywood Health Centre Chorleywood United Kingdom

**Keywords:** emergency medicine, telemedicine, health service research, eHealth, emergency, video consultation, remote, specialist, video conferencing, videoconferencing, video conference, telehealth, benefit, patient management

## Abstract

**Background:**

District general hospital emergency departments may refer patients to a tertiary center depending on the information available to a generalist clinician in discussion with a specialist team. If there is uncertainty, the lowest-risk strategy is often to transfer the patient. Video consultation allowing the specialist team to see and talk to the patient and local clinician while still in the emergency department could improve decision-making for patient transfer.

**Objective:**

The aim of this study is to assess the potential benefit of real-time video consultation between remote specialists and emergency department patients and clinicians across all specialties.

**Methods:**

Detailed patient data were collected prospectively for 6 months (between January 16, 2012, and July 15, 2012) on all patients presenting to a district general hospital emergency department who required input from a specialist team at the nearest tertiary care center. These patients were discussed retrospectively with the specialist teams to determine whether videoconferencing could have benefited their management. The logistics for the use of videoconferencing were explored.

**Results:**

A total of 18,799 patients were seen in the emergency department during the study period. Among the 18,799 patients, 413 referrals (2.2%) were made to the tertiary center specialist teams. A review of the patients transferred indicated that 193 (46.7%) of the 413 patients who were referred might have benefited from video consultation (193/18,799, 1% of all patients). If the specialist team could be accessed via videoconferencing only while a senior member was available in the hospital (8:00 AM-10:00 PM), then a maximum of 5 patients per week across all specialties would use the equipment. If 24-hour specialist access was available, this would increase to 7 patients per week.

**Conclusions:**

In regions where there is direct transportation of patients by ambulance to specialist centers and there is a regional picture archiving and communication system in place, video consultation between emergency department patients and specialists has limited potential to improve patient management.

## Introduction

Tertiary-level specialist input may be needed for patients in a district general hospital (DGH) emergency department (ED) to help with diagnosis or provide complex care beyond the capability of the DGH. Communication is usually done by telephone and decisions are based on reported history, examination, and preliminary investigations, often performed by a junior trainee. The specialist has few options for obtaining further reliable information at this stage and there is pressure to make a quick decision. The safest option is usually a patient transfer to the tertiary center, but that interrupts the continuous care of the patient, removes them from full resuscitation facilities during transfer, and isolates them from family and friends. The inappropriate transfer of patients also wastes resources in the tertiary center and ambulance service. There are other patients for whom the need for early specialist input may go unrecognized and, as a result, they would experience poor outcomes.

These difficulties in interhospital communication and patient transfer may be helped by the use of real-time videoconferencing (telemedicine) between the patient and local clinician at one end and the specialist clinician at the other. This allows the specialist to see the patient, ask focused questions relating to their condition, and observe specific aspects of the examination performed by the on-site clinician. This may confirm the need for transfer but allow it to be timed more appropriately, perhaps 1 day later, when the patient can be admitted directly to the specialist ward and treated on a planned elective list. Alternatively, it may lead to continued care in the secondary care hospital with advice from tertiary care. If specialist input can be accessed easily in the ED, then any patients for whom there is uncertainty in the decision to transfer could be assessed in this way with the aim of optimizing management.

Telemedicine, via videoconferencing, has been used to facilitate referrals between primary and secondary care, usually for the diagnosis and management of nonurgent conditions [1,2]. It has also allowed major EDs to provide support to smaller units in remote settlements and minor injury units [3-7]. Tertiary-level specialties have set up their own telemedicine services, particularly in cardiology and stroke medicine. The cardiology systems initially used the transmission of prehospital electrocardiograms performed by paramedics to enable early treatment with intravenous thrombolysis for myocardial infarction before arrival at the hospital [8]. This same system can now be used to divert an ambulance to a tertiary-level center to enable quicker percutaneous coronary revascularization and better outcomes [9-11]. Specialist stroke services have developed links with secondary care for early audiovisual patient assessment, with an aim of starting intravenous thrombolysis on-site within the 3-hour “therapeutic window” after a stroke [12-15]. These studies have shown that the audiovisual assessment of acute stroke compares well with conventional consultation [15]. Other areas where telemedicine has helped emergency care include plastic surgery [16,17], ophthalmology [18-20], and ear, nose, and throat (ENT) [21,22].

Single-specialty telemedicine links to a DGH may have a limited life expectancy if treatment protocol changes. It becomes redundant, for example, when patients who have had a stroke are transported directly from their homes to a tertiary center for intraarterial thrombolysis. Treatments change with time across all specialties and telemedicine is merely a tool to facilitate change. It will have an expanding role in some areas but a limited role in others. Within a DGH ED, therefore, telemedicine facilities are likely to be more useful if used flexibly across a range of conditions to communicate with specialist care clinicians. They would no longer be subject to the vagaries of a single specialty but could evolve with the development of techniques for aiding distant diagnosis, such as real-time ultrasound examination [23] and with new treatments which might be supported in a DGH.

The Horton General Hospital (HGH) ED transfers approximately 800 patients per year to specialties in Oxford. The Interhospital Telemedicine Study was designed to collect data on HGH ED patients requiring specialist input in the 6 months before and 6 months after the introduction of video consultation to assess the impact of the intervention. The analysis of the data from the first 6 months indicated that the impact would be minimal, and it was decided that the study should be terminated early. The results of the study are presented here.

## Methods

### Hospitals Involved in the Study

The HGH is a 277-bed DGH in Banbury, United Kingdom, serving the population of North Oxfordshire, South Warwickshire, and South Northamptonshire. It provides the following services: general medicine and surgery, cardiology (noninterventional), obstetrics and gynecology, and orthopedics; the HGH also has outpatient clinics but no full-time coverage for ophthalmology, plastic surgery, urology, and vascular surgery. It has a 2-bed intensive care unit. It is part of the Oxford University Hospital (OUH) Trust.

The Oxford services of the OUH are based in the John Radcliffe Hospital (JRH) and Churchill Hospital, 3 miles apart and 26 miles from Banbury. Most emergency provision is on the John Radcliffe site. The Oxford services provide tertiary-care services to the northern part of the South Central Regional Health Authority.

The Oxford and Banbury hospitals share a common picture archiving and communication system (PACS) so that radiological images from HGH can be readily viewed by Oxford clinicians. Local ambulance protocols are in place to take patients experiencing myocardial infarction or stroke directly from their homes to the OUH to ensure appropriate early intervention.

### Participants

All patients for whom specialist advice from the JRH was sought after assessment in the HGH ED, excluding those who had a clear diagnosis which required rapid transfer to the JRH, such as a ruptured abdominal aortic aneurysm, were included in this study.

### Ethics Approval

Ethics approval for the project was sought from the Oxfordshire Research Ethics Committee. The Research Ethics Committee advised us that the project was undertaking a service review rather than research and therefore permission was not required.

### Data Collected

A research manager and research nurse reviewed the notes of all patients about whom the JRH-based specialist team was contacted within 36 hours of the consultation between the HGH ED and the JRH-based specialist team. The research manager and research nurse collected the following data (see Multimedia Appendix 1 for the full data set):

- Age, sex, major comorbidities
- Date and time of the initial HGH ED assessment and transfer (if any) to JRH
- Next step in the patient pathway from the ED

Background data were collected over the same 6 months on the total number of patients seen in the HGH ED and the total number admitted to the HGH from the ED

### Data Analysis

Data were collected on the total number of presentations to the HGH ED, and admissions to the HGH and transfers to the JRH were collated under specialty headings with subgroups according to the nature of patient presentation within those groups.

### Focus Group Discussion

During and after the 6-month data collection period, discussions were held with the specialist teams who received the majority of referrals. These meetings included senior and junior medical staff and senior nurses and involved a review of the data collected on patients referred to each specialty to determine the reasoning and basis for the decision to transfer a patient or not. The meetings also aimed to determine the ways in which video consultation could have provided the potential benefit of teleconsultation.

The data on admissions to HGH were discussed with the HGH consultants most involved with this activity to determine if they perceived any potential advantage of teleconsultation in the ED before admission.

Discussions were also held with HGH ED staff and direct observations were made of activity in the department to understand the logistics of using teleconsultation.

## Results

### Patient Transfers

Between January 16, 2012, and July 15, 2012 (27 weeks), 18,799 patients were seen in the HGH ED. Of these 18,799 patients, 413 (2.2 %) were transferred to the JRH in Oxford and 3659 (19.5 %) were admitted directly to the HGH (Table 1 and Table 2).

Data on the specialties to which HGH admissions from the ED had occurred were analyzed for the first 3 months of this period. A total of 1539 patients were admitted to all specialties, with most being admitted to general medicine, general pediatrics, gerontology, and cardiology (Table 2).

**Table 1:** Data on transfers among patients from the Horton General Hospital emergency department to the John Radcliffe Hospital in Oxford, United Kingdom.

Specialty	Patients (n=413), n
Plastic surgery	98
Ophthalmology	88
Ear, nose, and throat	75
Pediatrics	39
Oral and maxillofacial surgery	32
Neurology	19
Vascular surgery	15
Urology	13
Stroke medicine	10
Cardiac medicine	9
Trauma	7
Renal medicine	2
Colorectal surgery	1
Gynecology	1
Miscellaneous	4

**Table 2:** The most common types of admission from the Horton General Hospital emergency department.

Specialty	Patients (n=1539), n (%)
General medicine	373 (24.2)
General pediatrics	213 (13.8)
Gerontology	162 (10.5)
Cardiology	142 (9.2)
Chest medicine	123 (8)
Others	526 (34.2)

### Focus Group Discussions

#### Specialty Groups

##### Plastic Surgery and Oral and Maxillofacial Surgery

Most transfers to these specialties were related to facial or hand fractures. There are protocols indicating which patients should be transferred, and most decisions were based on radiology images, which are accessed easily from both the HGH and Oxford.

Most facial lacerations were sutured at the HGH and there are guidelines on transfer. There appears to be no advantage to transmitting an image or holding a videoconference with the patient, except in the case of pediatric patients, for whom having an image would allow the surgical closure to be planned without disturbing the dressings beforehand. However, digital photographs can be taken and stored on Photoweb (Photoweb SAS) within the Trust in this situation. Occasionally images of intraoral lacerations might have assisted management decisions but generating high-quality images would be extremely difficult.

##### Ophthalmology

Most patients seen in the HGH ED had trauma to the front of the eye. There is a slit lamp available on-site and 3 ophthalmology clinics at the HGH during the week. Most patients were treated on-site and followed up with within 48 hours in-clinic if necessary. The few cases which needed transfer were discussed on the phone and the clinical opinion was that neither a direct detailed history from the patient (via a videoconference) nor video or still images would help in management decisions.

##### Ear, Nose, and Throat

Most transfers to ENT occurred because of persistent epistaxis. There appeared to be no advantage to videoconferencing or having further images sent from the HGH in these circumstances. Occasionally, patients presented with compromised airways at the HGH ED. These cases are best managed by the trained senior ED staff immediately available on-site and did not need immediate support from ENT specialists in Oxford, although some patients were transferred for subsequent care. Again, there seems to be no advantage to videoconferencing in these circumstances.

##### Pediatrics

Transfers to pediatric specialists occurred for a wide variety of problems, usually because the child required sedation and intubation. Videoconferencing provided no obvious advantage in these cases.

##### HGH Services

We also discussed with the HGH stroke physicians and cardiologists whether the management of patients they admitted to the HGH could be improved by videoconferencing from the ED. There are clear ambulance protocols for stroke and chest pain, which lead to most patients with a short history of stroke or myocardial infarction being transported directly to Oxford for emergency intervention. There are clear protocols for managing patients who are seen in the HGH ED. Very occasionally, there will be an unusual presentation of stroke, but this can usually be managed with phone advice from the JRH. There did not appear to be a role for videoconferencing here.

### HGH ED Staff

Concerns were raised over the nature of the hardware required for video consultation. The initial plan had been to use a trolley-mounted videoconferencing unit (Poly, Poly Inc) that could be moved to the patient’s trolley in the ED to provide the clinician at the JRH with a high-resolution image of the patient via a controllable unit with a mounted camera. It became clear from observation and discussion that moving such a large unit into a confined cubicle area would take time and effort and would be inconvenient for staff. It could only be justified if there was a clear and immediate benefit to patient management. We therefore looked at alternative technology for providing videoconferencing and considered tablets and smartphones. Unfortunately, using a small and easily portable device to improve the accessibility of videoconferencing also means that the device could be easily removed from the department, and there were concerns over equipment retention and the expenses of regular replacement

There were also concerns about developing familiarity with the teleconsultation system and making it a part of normal practice.

### Logistical Problems

The communication of clinical information must be secure and confidential. There is broadband communication between the HGH and Oxford-based hospitals, which can be used for videoconferencing. At present, we do not have the ability to extend this to clinicians outside the hospital (ie, at home). If we implemented videoconferencing with a specialist team in JRH, where the senior team members return to their homes at night, it is only likely to be effective (ie, involve a senior, experienced member of the team) during the day (possibly from 8:00 AM-10:00 PM).

We analyzed all 413 transfers to the JRH over 6 months. Only 46.7% (193/413) of transferred patients had a condition where videoconferencing or photography might have helped their management. Of the 413 transferred patients, 136 were seen between 8:00 AM and 10:00 PM and could potentially have benefited from videoconferencing. This is equivalent to only 5 patients per week across the range of specialties cited previously. If a video specialist link was available 24 hours per day, then 7 patients per week might have benefited from it. There are approximately 12 middle-grade physicians employed in the HGH ED between 8:00 AM and 10:00 PM each week; therefore, over the course of 6 months, each physician would see 13 patients (1 every other week) for whom videoconferencing or imaging might support specialist advice from the JRH. It seems unlikely that these physicians would consider using videoconferencing with so few cases per physician. The specialist team at the JRH could ask for videoconferencing but, again, it would be used only occasionally by each individual within the specialist team. Unless an intervention is used regularly by the individuals involved, with some tangible benefit to its use, it seems unlikely that it would be used at all.

## Discussion

### Principal Findings

This study has shown that in a well-run DGH ED with regular links to a tertiary center, the common presentations which require specialist advice can be managed with the use of protocols regarding patient transfer, supplemented by radiological image transmission (PACS) and image management. This study has also shown that, contrary to expectation, a specialist history and/or real-time patient imaging is rarely critical in deciding on management. Redesigning patient pathways from home, so that ambulances go directly to a major center for certain categories of patients has also had a major impact on delivering patients to the appropriate location for assessment and treatment.

### Comparison With Prior Work

The data indicate that potential opportunities to use telemedicine for referral are few in a DGH (up to 7 per week), and at such low levels, it is unlikely that the required skills would be maintained or that use would persist. This matches findings from other studies; one review found relatively few EDs (146 out of 4507 respondents) in the United States were using telemedicine for the transfer of patients [24]. Other reviews indicate that the majority of reports on the use of telemedicine for transfer in emergency medicine are from pilot projects that report favorably on user experience during the project, but there is no assessment of the potential for use [25]. Further reviews of the use of telemedicine in EDs conclude that video consultation has significant potential but there is still a lack of evidence supporting improved patient outcomes [26] and feasibility [27].

However, we have been unable to find any reports providing an audit of the potential for the use of telemedicine in making referrals from a DGH ED to a tertiary care center for the comparison of results.

### Future Directions

Changes in patient management in the future may create opportunities for the use of videoconferencing between patients in the ED and specialist teams, but systems need to be developed that combine ease of access with security measures to retain equipment. The cost-effectiveness of any such intervention would rapidly decline if tablets or phones had to be frequently replaced. Such systems should also seek to improve specialist access to videoconferencing through the flexible use of different devices (eg, smartphone, tablet, desktop PC, or laptop) in different sites (operating room, office, home) to allow 24-hour service provision and the engagement of clinicians.

### Limitations

The study has the limitation that it was conducted in a single DGH in England and was therefore influenced by local policies for emergency admission (eg, patients known to have experienced a stroke are taken directly to a specialist stroke center), and the distance between the hospitals was relatively small. However, experiences will vary in areas with different local policies for referral and different influences of geography on location and distance between hospitals.

### Conclusions

We have collected detailed information on the patients transferred from a DGH ED to a specialist center and admitted locally over a 6-month period. We have used that data to explore the potential for the use of videoconferencing with the specialists and local clinicians involved. Additionally, we have shown that the use of videoconferencing between patients and specialists in regions where there is a policy of direct transportation of patients by ambulance to specialist centers as well as a regional PACS has limited potential when used for the common presentations in a DGH ED. Further research is required to determine the potential for use in other locales.

## Appendix

Multimedia Appendix 1 Data collected on each patient for whom specialist advice was sought.

## Abbreviations

DGH: district general hospital
ED: emergency department
ENT: ear, nose, and throat
HGH: Horton General Hospital
JRH: John Radcliffe Hospital
NIHR: National Institute for Health and Care Research
OUH: Oxford University Hospital
PACS: picture archiving and communication system
