# Supplementary material for: An Assessment of the Potential Benefits of Video Consultation in the Emergency Department: Mixed Methods Study
Source: Interact J Med Res. 2022 Sep 15;11(2):e36081. doi: 10.2196/36081 (PMC9523531; doi:10.2196/36081)
Supplement: Multimedia Appendix 1 [file ijmr_v11i2e36081_app1.doc]

# Appendix 1

Data collected on each patient for whom specialist advice was sought:

1. Age, sex, major comorbidities
2. Date and time of initial HGH ED assessment and of transfer (if any) to Oxford
3. Next step in patient pathway from ED.

- transfer immediately to Oxford,
- admit HGH,
- admit HGH with plan for later transfer to Oxford,
- discharge with arrangement for Oxford elective admission,
- discharge with arrangements for Oxford OPD appointment,
- discharge with HGH follow up (elective admission or OPD),
- discharge with no follow up,
- died in HGH ED

1. Pathway after initial assessment in Oxford

- discharge with no inpatient treatment and no OPD follow up
- discharge with OPD follow up at HGH
- discharge with OPD follow up in Oxford
- immediate return to HGH
- admitted for observation, investigation or treatment
- died in transit or before intervention possible

1. If patient remains under care of HGH was there subsequent transfer to Oxford in the same episode of care?
2. Length of stay in Oxford and at HGH
3. Died after admission to HGH or Oxford?
